# Investigation of the efficiency of different reprocessing methods on disposable laryngeal masks contaminated with HBV DNA

**DOI:** 10.3205/dgkh000524

**Published:** 2024-12-16

**Authors:** Günhan Gökahmetoğlu, Duygu Perçin Renders, Selma Gökahmetoğlu, Cihangir Biçer, Recep Aksu, Şerife Çevik

**Affiliations:** 1 Department of Anesthesiology, Kayseri Training and Research Hospital, University of Health Sciences, Kayseri, Turkey; 2 Department of Medical Microbiology, School of Medicine, Kutahya Health Sciences University, Kutahya, Turkey; 3 Erciyes University, Faculty of Medicine, Medical Microbiology Dept., Kayseri, Turkey; 4 Erciyes University, Faculty of Medicine, Anesthesiology and Reanimation Dept., Kayseri, Turkey; 5 Uzun Mehmet Chest and Work Diseases Hospital, Medical Microbiology Dept, Zonguldak, Turkey

**Keywords:** laryngeal mask, sterilization, thermal disinfection, disinfection with peracetic acid, HBV DNA

## Abstract

**Background:**

The use of laryngeal masks (LM) has increased widely today, both in anesthesia and in emergency cases. LM are available as reusable and disposable. Although reuse of disposable LM is not recommended, they are reused again after decontamination with different methods in anesthesia units in some countries. The reprocessing of single-use LM was therefore investigated. The hepatitis B virus (HBV), hepatitis C virus (HCV), and human immunodeficiency virus (HIV) are pathogens that can pass into saliva. It is known that the HBV is more resistant to decontamination methods as compared to HCV and HIV.

**Objectives:**

In this study, it was aimed to investigate the effectiveness of different decontamination methods on disposable and reusable LM and to evaluate the reusability of disposable LM after they were treated with simulated saliva samples containing HBV DNA.

**Study design and setting:**

The observational study was carried out in Medical Microbiology Department of Erciyes University Medicine Faculty between March 2016 and Mach 2018.

**Method:**

Simulated saliva samples were prepared, and plasma samples of patients with plasma HBVDNA levels of 10^8^IU/mL were inoculated into these samples. HBV DNA levels in saliva samples were investigated by the real-time PCR (Qiagen, Germany). Reusable and disposable LMs were placed into HBV DNA-positive simulated saliva samples. The LM were kept in saliva at 37°C for 1 hour, then dried for 24 hours at room temperature. After cleaning in the automatic washer, different decontamination methods were applied to the LMs. Decontamination methods applied to reusable and disposable LM were thermal disinfection 1 minute at 90°C (A_0_600), thermal disinfection 5 minutes at 90°C (A_0_3000), thermal disinfection (A_0_600) + hydrogen peroxide gas plasma sterilization, thermal disinfection (A_0_600) + ethylene oxide sterilization, and disinfection with high-level disinfectant with 2% peracetic acid without cleaning in the automatic washer. Also, thermal disinfection (A_0_3000) +5 minutes steam sterilization at 134°C was implemented only to reusable LM. At least three LM were used for each group. Control samples were also used. After the decontamination procedures, the LM were kept in phosphate buffer (PBS) solution for 1 hour at 37°C with shaking. The presence of HBV DNA was investigated by the real-time PCR by taking samples from PBS. The polyethylene glycol procedure was used for saliva and nucleic acid isolation. After the decontamination procedures, the functioning control of the LM was controlled.

**Results:**

The HBV DNA level in the simulated saliva samples was 100,000 IU/mL (lg 5). No HBV DNA was detected in reusable and disposable LM after A_0_600 thermal disinfection + ethylene oxide and A_0_600 thermal disinfection + hydrogen peroxide. No HBV DNA was detected in reusable LM after A_0_3000 thermal disinfection+ steam sterilization. However, HBV DNA was detected in LM after A_0_600 or A_0_3000 thermal disinfection alone and disinfection with peracetic acid.

While no deformation was observed in reusable LM after reprocessing, deformation was observed in disposable LM.

**Conclusion:**

Reuse with high-level disinfection, which is frequently applied to disposable LM, is an incorrect and dangerous practice, therefore disposable LM should not be reused. Reusable LM can be reused after being reprocessing with thermal disinfection + steam or thermal disinfection + hydrogen peroxide low temperature sterilization after effective cleaning.

## Zusammenfassung

**Hintergrund:**

Die Verwendung von Larynxmasken (LM) hat heute sowohl in der Anästhesie als auch bei Notfällen stark zugenommen. LM sind als wiederverwendbare und als Einwegmasken erhältlich. Obwohl die Wiederverwendung von Einwegmasken nicht empfohlen wird, werden sie in einigen Ländern nach der Aufbereitung mit verschiedenen Methoden in Anästhesieeinheiten erneut verwendet. Daher wurde die Aufbereitung von Einweg-LM untersucht. Das Hepatitis-B-Virus (HBV), das Hepatitis-C-Virus (HCV) und das humane Immundefizienz-Virus (HIV) sind Erreger, die in den Speichel übergehen können. Es ist bekannt, dass das HBV im Vergleich zu HCV und HIV resistenter gegen Desinfektionsmethoden ist.

**Zielsetzung:**

Es sollte die Wirksamkeit verschiedener Dekontaminationsmethoden für Einweg- und Mehrweg-LM untersucht und die Wiederverwendbarkeit von Einweg-LM nach der Behandlung mit simulierten Speichelproben, die HBV-DNA enthalten, bewertet werden.

**Studiendesign und Setting:**

Die Beobachtungsstudie wurde in der Abteilung für medizinische Mikrobiologie der Medizinischen Fakultät der Erciyes Universität zwischen März 2016 und Mach 2018 durchgeführt.

**Methode:**

Es wurden simulierte Speichelproben hergestellt und Plasma von Patienten mit HBV-DNA-Werten von 10^8^ IU/mL im Plasma in die Proben übertragen. Die HBV-DNA-Werte in den Speichelproben wurden mittels Real-time-PCR (Qiagen, Deutschland) untersucht. Mehrweg- und Einweg-LM wurden 1 h bei 37°C im Speichel gelagert und dann 24 h bei Raumtemperatur getrocknet. Nach der Reinigung in einem Reinigungs- Desinfektions-Gerät wurden die LM mit verschiedenen Dekontaminationsmethoden behandelt. Bei den Dekontaminationsmethoden für Mehrweg- und Einweg-LMs handelte es sich um eine 1-minütige thermische Desinfektion bei 90°C (A_0_600), eine 5-minütige thermische Desinfektion bei 90°C (A_0_3000), eine thermische Desinfektion (A_0_600) + Wasserstoffperoxid-Gasplasma Sterilisation, eine thermische Desinfektion (A_0_600) + Sterilisation mit Ethylenoxid und eine Desinfektion mit einem high-level Desinfektionsmittel mit 2% Peressigsäure ohne vorherige Reinigung. Die thermische Desinfektion (A_0_3000) + 5 min Dampfsterilisation bei 134°C wurde nur bei Mehrweg LM durchgeführt. Für jeden Versuch wurden mindestens drei LM verwendet. Ebenso wurden Kontrollen verwendet. Nach der Aufbereitung wurden die LM für 1 h in Phosphatpufferlösung (PBS) bei 37°C unter Schütteln aufbewahrt. Das Vorhandensein von HBV-DNA wurde mittels Real-Time-PCR untersucht, indem Proben aus PBS entnommen wurden. Für die Speichel- und Nukleinsäureisolierung wurde das Polyethylenglykolverfahren verwendet. Nach der Aufbereitung wurde die Funktionskontrolle der LM durchgeführt.

**Ergebnisse:**

Die HBV-DNA-Konzentration in den simulierten Speichelproben betrug 100.000 IU/mL (lg 5). Es wurde keine HBV-DNA in Mehrweg- und Einweg-LM nach A_0_600 thermischer Desinfektion + Ethylenoxid und A_0_600 thermischer Desinfektion + Wasserstoffperoxid nachgewiesen. Auchbei Mehrweg-LM wurde nach thermischer Desinfektion A_0_3000 + Dampfsterilisation keine HBV-DNA nachgewiesen. Allerdings wurde HBV-DNA in LM nach alleiniger thermischer Desinfektion mit A_0_600 oder A_0_3000 und nach Desinfektion mit Peressigsäure nachgewiesen.

Während bei wiederverwendbaren LM nach der Aufbereitung keine Verformung festgestellt wurde, war das bei Einweg-LM der Fall.

**Schlussfolgerung:**

Die Wiederverwendung nach high-level Desinfektion, die häufig bei Einweg-LM angewendet wird, ist eine falsche und gefährliche Praxis, so dass Einweg-LM nicht wiederverwendet werden sollen. Mehrweg-LM können wiederverwendet werden, nachdem sie mit thermischer Desinfektion + Dampf oder thermischer Desinfektion + Wasserstoffperoxid Niedrigtemperatur-Sterilisation nach einer wirksamen Reinigung aufbereitet wurden.

## Introduction

The laryngeal mask (LM) is an essential airway tool developed after the endotracheal tube. LMs are widely used not only by anesthetists in the operating room but also by paramedics or other healthcare personnel during emergency interventions [1].

Disposable and reusable LM are available in the market, and reusable LM are preferred as they are economical. During routine use, the oral secretions of patients contaminate LM. Therefore, pathogens that pass into the saliva can be transmitted from one patient to another through LM. This situation highlights the importance of the reprocessing methods applied to LM.

Hepatitis B virus (HBV), hepatitis C virus (HCV), and human immunodeficiency virus (HIV) are viruses that can pass into saliva. It is known that HBV is more resistant to decontamination methods as compared to HCV and HIV [2]. In addition, studies have shown that HBV DNA is also positive in the saliva of patients with a blood HBV DNA level of 10^6^ copies/mL and above [3], [4].

Reusable LM are reprocessed and reused after being used. Disposable LM are also reused in some hospitals or emergencies in some countries. Different reprocessing methods are applied in different hospitals for LM. After applying different methods to reusable and disposable LM, it is important to determine whether LM can be used again in different patients. Therefore, it was aimed to investigate the effectiveness of different reprocessing methods and to evaluate the reusability of disposable LM after they were treated with simulated saliva samples containing HBV DNA.

## Material and method

### Ethics

Ethical approval of this study (Ethical Committee No 2014/100) was provided by the Clinical Research Ethics Committee of Erciyes University, Kayseri, Turkey (Chairperson Prof. Dr. R. Düsünsel) on 21 February 2014.

### Study performance

The study was carried out in Medical Microbiology Department of Erciyes University Medicine Faculty between March 2016 and Mach 2018. 

The blood samples containing 10^8^ IU/mL HBV DNA among all samples received by Erciyes University Faculty of Medicine Medical Microbiology Department Virology Laboratory were kept in a deep freezer at –70°C until the day of the study. The content of simulated saliva was prepared as stated in the literature, consisting of 1.09 mmol/L CaCl_2_, 0.68 mmol/L KH_2_PO_4_, 30 mmol/L KCl, and 2.6 mmol/L NaF [5]. Adjustment of the simulated saliva pH to 7.4 was achieved with 50 mmol/L Hydroxyethyl piperazine ethanesulfonic acid (Hepes). A 2 mL serum sample with a plasma HBV DNA level of 10^8^IU/mL was added into the prepared simulated saliva and mixed well. HBV DNA level was investigated using a real PCR method (Rotorgene Qiagen, Germany) in 1 mL of this simulated saliva sample.

### Determination of HBV DNA

Nucleic acid extraction from plasma samples was performed in the QIAsymphony SP device using the extraction kit (QIAsymphony DSP Virus/Pathogen Midi Kit, QIAGEN, Germany) per the manufacturer's recommendations. HBV DNA amplification and quantification were performed per the manufacturer’s recommendations for detecting and quantifying HBV DNA, using the Artus HBV QS-RGQ Kit (QIAGEN, Germany) and RT-PCR device (Rotor-Gene Q, Corbett Research, Austria). This diagnostic test kit specifically amplifies the 134 bp core region of the HBV genome. In each study, the presence of PCR products was determined by increasing the amount of fluorescence using five quantitation standards and a negative control sample. The analytical sensitivity of the test is 10.2 IU/mL, and the linear range is 31.6–2x10^7^ IU/mL.

The HBV DNA level in the simulated saliva was found to be 10^5^ IU/mL, and different reprocessing methods were applied to the LM after they were placed in the simulated saliva samples containing HBV DNA. The LM were kept in saliva at 37°C for 1 hour, then dried for 24 hours at room temperature. After cleaning in the automatic washer Belimed, (Switzerland) with a program including prewash (1 minute in cold water), main wash using alkaline detergent 10 minutes at 45°C, rinsing (cold water), neutralization using phosphoric acid and rinsing with (deiyonized water), different decontamination methods were applied to the LM.

### Reprocessing and success control

Reusable LM were reprocessed accordingly:

- Thermal disinfection at 90°C for 1 minute (A_0_600) resp. at 90°C for 5 minutes (A_0_3000)
- Thermal disinfection (A_0_600) + hydrogen peroxide sterilization (STERRAD 100 X, ASP, USA)
- Thermal disinfection (A_0_600) + ethylene oxide sterilization (3M, USA)
- Thermal disinfection (A_0_3000) + 5 minutes steam sterilization at 134°C
- Disinfection with 2% peracetic acid without pre-treatment in the automatic washer.

Reprocessing of disposable LM were performed accordingly:

- The methods are the same as those mentioned above except thermal disinfection (A_0_3000) + 5 minutes steam sterilization at 134°C.

Three LM were used for each group. In addition, control LM samples were also used three per in the study. After the decontamination procedures, the functioning of the LM was controlled.

Following the reprocessing, the LM were kept in sterile PBS solution for 1 hour at 37°C by shaking.

The polyethylene glycol precipitation method was used to show the presence of HBV DNA in PBS [6]. HBV DNA level was investigated by PCR method and real PCR method (Qiasymphony Qiagen, Germany).

## Results

The mean HBV DNA level in the simulated saliva samples was 100,000 IU/mL (lg 5).

HBV DNA was not detected in LM after application of A_0_600 + ethylene oxide and A_0_600 + hydrogen peroxide, as well as in reusable LM after steam sterilization. However, HBV DNA was detected with all other decontamination methods (Table 1 (Tab. 1)).

While no deformation was observed in reusable LM after reprocessing, deformation was observed in disposable LM after decontamination processes.

## Discussion and conclusion

The fact that LM can be applied to patients quickly and with high success and that users can provide airway patency without intensive training has increased the frequency of use of these devices. Currently, endotracheal intubation or LM are used in the majority of patients for airway management in out-of-hospital cardiac arrests in the United States [7] . 

The types of LM used include reusable and disposable. Disposable LM are made of polyvinyl chloride (PVC), reusable LM are primarily made of silicone [8]. The substance called "diethyl hexyl phthalate" (DEHP), which provides flexibility in PVC-based products, has brought some concerns. The Environmental Protection Agency classifies DEHP as a possible carcinogen [9]. In the recommendations published by the Food and Drug Administration (FDA) in 2002, the necessity of reducing exposure to DEHP was emphasized [10]. These concerns with PVC do not apply to reusable LM made of silicone [8]. 

Reusable LM are preferred as they are economical and sustainable. Various reprocessing methods are applied to reusable LM after use. Since reusable LM are semi-critical instruments, in the European Union a high-level disinfection is required [11]. However, it is reported in the literature that the LM becomes unusable after autoclave sterilization [12]. It is known that the PVC material in disposable LM absorbs disinfectant active agents [13]. Therefore, it is recommended to use medical materials made of silicone [14]. 

HBV infection is a critical health problem affecting 10% of the world's population. According to the WHO report, more than 257 million people are chronically affected by this virus throughout their lives [15]. 

HBV, HCV, and HIV are viruses that can pass into saliva. HBV passes into bodily fluids, such as blood, saliva, tears, semen, and vaginal secretions. As it is known, the HBsAg positivity rate in our country is 2–8%, while anti-HCV positivity is around 1% [16], [17]. It is known that HBV is more resistant to sterilization methods as compared to HCV and HIV. Although HBV is an enveloped virus, it is not inactivated by detergents like other enveloped viruses [2]. When any instrument is contaminated with blood containing HBV, the contagiousness of HBV continues for >1 week [18], [19]. HBeAg-positive individuals have 10^8^–10^10^ virion/mL virus particles in their serum among HBV-infected individuals. The intraoral fluid of the individual is essentially the ultrafiltrate of the plasma [20]. Studies have shown that HBV DNA is also positive in the saliva of patients with a blood HBV DNA level of more than 10^6^ copies/mL [3], [4]. In some studies, the detection of HBsAg in saliva has been evaluated, and it has been shown that saliva can be used as an alternative sample to serum for identifying HBsAg carriers [21], [22], [23]. However, it is known that HBsAg cannot be detected when the HBV DNA level in the blood is below 10^3^ IU/mL [24]. Hence, different decontamination methods were applied in our study after treating LM with simulated saliva samples contaminated with HBV DNA. 

In the pre-anesthetic evaluation report updated by the American Society of Anesthesiology (ASA), it is not recommended to measure hepatitis indicators in routine tests before surgery [25]. Therefore, in patients requiring out-of-hospital or in-hospital cardiopulmonary resuscitation (CPR), LM should be used when airway management is so vital that it competes with minutes. It cannot be questioned whether the patients using this device have hepatitis due to the urgency of airway provision. 

The study of Parker et al. [26]. found the rate of contamination with occult blood in LM to be 76%, while the rate of contamination with visible blood was 12%. During routine use, the oral secretions of patients contaminate LM. Therefore, pathogens that pass into the saliva can be transmitted from one patient to another through LM. If the different reprocessing methods applied to the LM are insufficient, pathogens can be transmitted from patient to patient, as well as pathogens can be transmitted to healthcare personnel during the application of LM.

Proteins and lipids in blood show a very high affinity for silicone materials. They tend to form a thin biological film layer several nanometers thick on the surface of the LM [27]. In studies conducted, after routine cleaning and sterilization of reusable LM, protein residues were detected in LMs [28], [29], [30]. Therefore, one of the main concerns with reuse is that these biological residues cannot be adequately cleaned, posing a risk of cross-contamination. It demonstrates the importance of evaluating current sterilization methods applied to reusable LM to control diseases that can spread due to protein residues [27]. Besides, in the study of Richard et al. [31] when used LM were reprocessed together with unused LM, protein residues were detected, even in unused LM. This situation highlights the importance of effective reprocessing methods applied to LM. 

In the literature, three of six studies on protein contamination of LM have shown that all reusable LM have protein contamination after conventional cleaning and autoclaving procedures recommended by the manufacturer [28], [29], [30]. In two studies where additional cleaning procedures, such as ultrasonic cleaning [32] and washing with potassium permanganate 2 mg/L [33], were applied, it was reported that protein contamination decreased. In another study, protein contamination was demonstrated in LM, despite the addition of automatic machine washing to the cleaning procedure [34]. 

Lee et al. [35] investigated HBV DNA before washing, after washing, and after disinfection (with glutaraldehyde or electrolyzed acid solution), after combining endoscopes with HBV- positive sera in their study. They found HBV DNA negativity only in endoscopes after disinfection. In the same study, HBV DNA was found before and after washing. In another study, it was determined that, as a result of keeping the endoscopes with peracetic acid at a concentration of 0.065% for 1 hour, severe damage to the protein and genome structure of HBV occurred, and there was a decrease of at least 4 lg in virus infectivity [36]. 

Disposable and reusable LM are available in the market, latter are reused after reprocessing. Although not recommended in some cases, disposable LM are used again after the application of different reprocessing methods. The standard method for reprocessing reusable LM is washing + thermal disinfection + steam sterilization [11], [37]. 

However, due to the scarcity of materials and the intense case tempo in hospitals, reusable LM are only disinfected before use. When the literature was examined, no article was found with regard to reprocessing disposable and reusable LM with different methods after exposing them to HBV-positive sera. After applying different reprocessing methods to reusable and disposable LM, placed in HBV DNA-positive simulated saliva samples, this study evaluated for the first time, whether LM could be used in different patients.

HBV DNA was not detected in reusable and disposable LM after application of A_0_600+ethylene oxide and A_0_600+hydrogen peroxide. Additionally, HBV DNA was not detected in reusable LM after steam sterilization. However, HBV DNA remained with all other reprocessing methods. Also, deformation was not observed in reusable LM after reprocessing, while deformation was observed in disposable LM after decontamination. Thus, it was also shown that the high-level disinfection and “reuse” practice frequently applied to disposable LM is incorrect. Disposable LM should not be reused. Reusable LM can be reused after being sterilized with thermal disinfection + steam or thermal disinfection + other low temperature sterilization methods after effective cleaning. In this way, transmission of HBV and other pathogens between patients can be prevented by the use of reusable LM.

## Notes

### Authors’ ORCID

- Günhan Gökahmetoglu: 0000-0001-8281-2667
- Duygu Perçin Renders: 0000-0002-4436-5226
- Selma Gökahmetoglu: 0000-0002-7747-6045
- Cihangir Biçer: 0000-0001-8163-6681
- Recep Aksu: 0000-0001-7825-2134
- Serife Çevik: 0000-0002-4631-289X

### Ethical approval

All applications were realized under the approval of Erciyes University Ethics Committee.

### Fundig

This study was supported by Erciyes University Scientific Research Projects Coordination Unit. ERÜ/BAP, Project No: (TCD-2015-5721). Support was provided solely from institutional and departmental sources

### Competing interests

The authors declare that they have no competing interests. 

## Figures and Tables

**Table 1:**
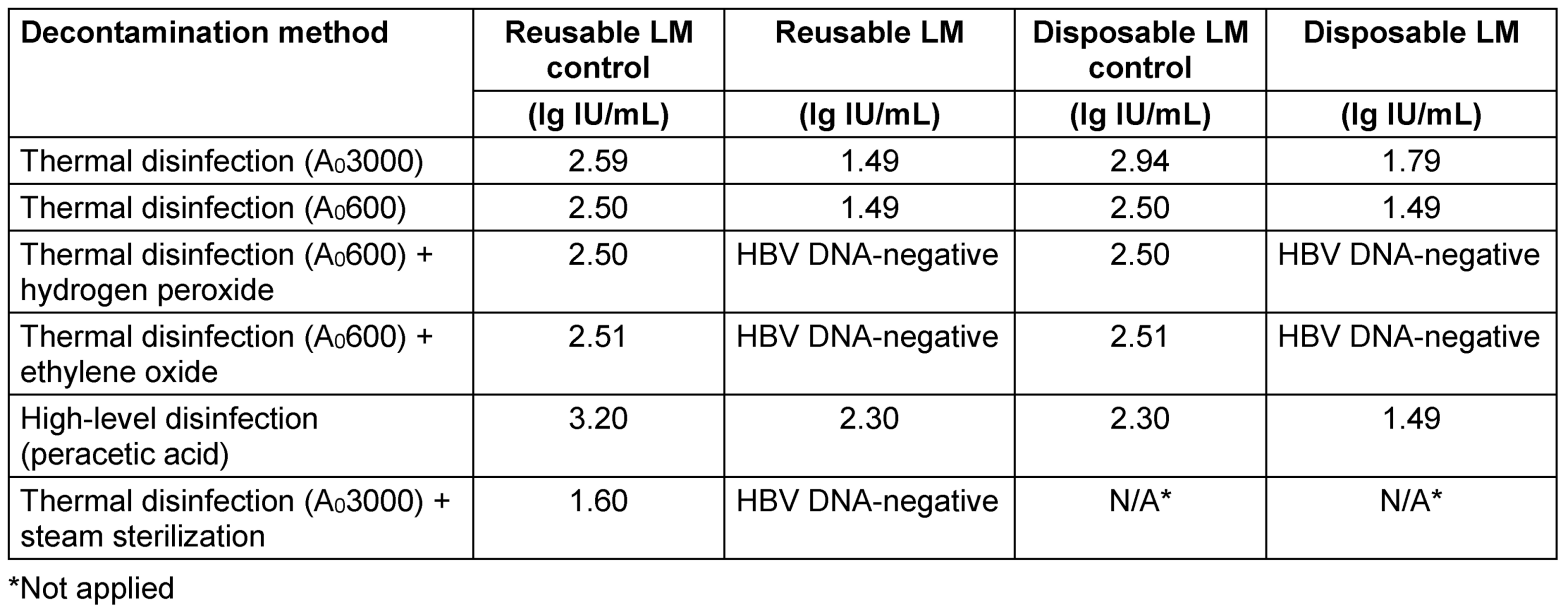
HBV DNA results obtained with different reprocessing methods
